# Alterations of Gut Microbiome in the Patients With Severe Fever With Thrombocytopenia Syndrome

**DOI:** 10.3389/fmicb.2018.02315

**Published:** 2018-10-01

**Authors:** Honghai Xu, Yuanyuan Wei, Hongqiu Ma, Yanyan Liu, Yalong Zhang, Lifen Hu, Jiabin Li

**Affiliations:** ^1^Department of Infectious Diseases, The First Affiliated Hospital of Anhui Medical University, Hefei, China; ^2^Department of Pathology, The First Affiliated Hospital of Anhui Medical University, Hefei, China; ^3^Anhui Center for Surveillance of Bacterial Resistance, Hefei, China; ^4^Department of Hospital Infection Control, The First Affiliated Hospital of Anhui Medical University, Hefei, China

**Keywords:** sever fever with thrombocytopenia syndrome (SFTS), gut microbiome, 16S rDNA sequencing, *Bacteroidetes*, *Firmicutes*, *Proteobacteria*, clinical symptoms, key serum enzymes

## Abstract

Severe fever with thrombocytopenia syndrome (SFTS) is an emerging tick-borne infectious disease caused by SFTS virus, and the number of SFTS cases increased year by year in China. Previous studies had indicated that gut microbiome closely associated with human health and diseases, including infection diseases, liver diseases, gastrointestinal diseases and metabolic diseases. The aim of this study is to investigate the alterations and involvements of gut microbial in SFTS patients. We compared the gut microbiome of 26 SFTS patients between 20 health controls using the Illumina MiSeq sequencing platform. Reduced gut microbiota diversity and dramatic shifts of fecal microbial composition in SFTS patients were observed compared with health controls. In the intestinal microbial of SFTS patients, the *Lachnospiraceae* and *Ruminococcaceae* which could produce short-chain fatty acids were clearly dropped compared with health people, meanwhile, *Sutterella* which have anti-inflammation properties were reduced too. On the contrary, some common opportunistic pathogens like *Enterococcus* and *Streptococcus* and endotoxin-producing bacteria *Escherichia* which could rise the risk of infections were increased in SFTS patients than healthy people, in addition lactate-producing bacteria *Lactobacillaceae* also significantly increased in SFTS patients. In addition, research findings on the correlation between gut microbiota and biochemical data found that the changes of gut microbiota of SFTS patients were closely associated with clinical symptoms, key serum enzymes, infection and mortality. These alterations of gut microbiome in SFTS patients suggest the potential contributions of gut microbial to the pathogenesis of SFTS.

## Introduction

Sever fever with thrombocytopenia syndrome (SFTS) is a recent confirmed tick-borne disease caused by the severe fever with thrombocytopenia syndrome virus (SFTSV), the virus which is classified in the family *Phenuiviridae*, genus *Phlebovirus* (Yu et al., 2011). In 2009, the SFTSV was first identified in the rural area in central China, and after then although SFTS cases had been reported in other East Asia, including Japan (Kurihara et al., 2016) and Korean (Park et al., 2016), the most SFTS cases were reported in China. From 2011 to 2016, the annual case numbers increased year by year in China (Sun et al., 2017), SFTS has already posed as a severe threat to public health. The clinical symptoms of SFTS include fever, lymphadenopathy, vomiting, diarrhea, anorexia, headache, fatigue, chill, myalgia, abdominal pain, gingival hemorrhage and so on (Sun J. et al., 2014). The severe cases could present with hemorrhagic, neurologic, multiple organs dysfunction, and even developing fatal outcome (Sun Y. et al., 2014). Although recently studies had reported that dysfunction of innate immune response (Song et al., 2017), cytokine storm and endothelial activation/dysfunction (Li et al., 2017) were associated with SFTS, the pathogenesis of SFTS has not been fully defined.

Gut microbiota, act as a real organ, the interactions between gut microbial and multiple diseases had received considerable attention. Previous literature had confirmed that the dysfunction of gut microbiome was closely related to the liver diseases, including liver cirrhosis (Chen et al., 2011; Bajaj et al., 2014), liver cancer (Yoshimoto et al., 2013), non-alcoholic steatohepatitis (Zhu et al., 2013). And the alterations of intestinal microbial in human diseases, such as obese (Turnbaugh et al., 2009; Henao-Mejia et al., 2012), inflammatory bowel disease (Palm et al., 2014), colorectal cancer (Gagnière et al., 2016), diabetes mellitus (Qin J. et al., 2014) are becoming increasing clear through numerous studies.

It is noteworthy that no study had focused on the potential role of gut microbiome played in the pathogenesis of SFTS. The alterations and involvements of gut microbiome in SFTS are still not clear, and elucidating the characteristics of the gut micobiome in SFTS patients will help unravel the detailed pathogenic role of gut dysbiosis in the pathogenesis of SFTS. Thus, the aim of this study was to investigate the changes in gut microbial of SFTS patients. Here, to fulfill the research purpose, we used the Illumina MiSeq high-throughput sequencing platform targeting the 16S rDNA gene, to compare the fecal microbial communities in SFTS patients with health people. And a comprehensive study was conducted on the correlation between clinical manifestations, key serum enzymes, infection and gut microbiota in SFTS patients.

## Materials and Methods

### Patients Information

The study was approved by the Ethical Review Board of the first affiliated hospital of Anhui Medical University and conformed to the ethical guidelines of the 1975 Declaration of Helsinki. All subjects obtained informed consent and all experiments were performed in accordance with relevant guidelines and regulations. The 26 SFTS patients who were included in were had been treated in the first affiliated hospital of Anhui Medical University during 2016–2017, and the 20 health volunteer subjects were randomly recruited during their annual health survey in the same hospital. The health controls have comparable age and gender with SFTS patients. And all subjects from the same mountain areas, the Ta-pieh Mountains (**Supplementary Table S1**), which located in southwest of Anhui Province. For all subjects, the exclusion criteria included the following: gastrointestinal disease, a history of cancer, diabetes mellitus, liver diseases, inflammation, others infectious diseases, intake history of any medicine, antibiotics or probiotics within the preceding 3 months. The SFTS patients was confirmed by real-time reverse transcription-polymerase chain reaction (RT-PCR) tests or serological tests as guided by the Ministry of Health, China (Ministry of Health China, 2011). The baseline characteristics of subjects are shown in **Table 1**.

**Table 1:** Baseline characteristics of subjects.

	HC^a^	SFTS^a^	*P*-value
Age^b^ (mean ± SD)	55.35 ± 10.30	59.73 ± 9.03	0.13
Male/female	9/11	13/13	0.74
ALT^c^ (IQR)	23.50 (10.25–26.00)	81.50 (51.00–268.00)	<0.01
AST^c^ (IQR)	23.00 (21.00–27.25)	162.00 (85.50–362.00)	<0.01
LDH^c^ (IQR)	199.00 (180.50–222.50)	1159.00 (841.75–2150.00)	<0.01
CK^c^ (IQR)	80.50 (71.50–107.75)	234.00 (134.25–838.25)	<0.01

*^a^SFTS, severe fever with thrombocytopenia syndrome; HC, health control. ^b^Date are expressed as the mean ± SD, Independent t-test was used to test for significant difference among two groups (P < 0.05). ^c^ALT, alanine transaminase, the normal range is 21–72 U/L; AST, aspartate transaminase, the normal range is 17–59 U/L; LDH, lactate dehydrogenase, the normal range is 313–618 U/L; CK, creatine kinase, the normal range is 55–170 U/L. Data are expressed as the Inter-Quartile Range (IQR), Mann–Whitney U test was used to test for significant difference among two groups (P < 0.05).*

### Sample Collection

Each SFTS patient and healthy volunteer provided a fresh stool sample that was delivered immediately from our hospital to the laboratory, and it was divided into three aliquots of 200 mg and immediately stored at -80°C. For SFTS patients, an additional blood sample were required. 5 mL of blood was collected immediately in gel-separator vacuum test tubes (Hubei Jingxing Tech Co. Ltd., Hubei, China) from SFTS patients who was admitted to the hospital. All samples were kept at room temperature for 30 min until clotted. All blood samples were centrifuged to separate the serum at 1200 *g* for 10 min within 2 h after collection, and serum was analyzed within 2 h after separation.

### DNA Extraction

DNA was extracted from 200 mg of each stool sample using a QIAamp DNA Stool Mini Kit (Qiagen, Hilden, Germany) following the manufacturer’s instructions. The amount of DNA was measured by NanoDrop (Thermo Scientific) and its molecular size was estimated by agarose gel electrophoresis. All DNA was stored at -20°C until further analysis.

### 16S rDNA V4 PCR Amplification and MiSeq Sequencing

PCR amplification of the V4 hypervariable region of the16S rDNA gene was performed using the universal bacterial primers 515F(5′-GTGCCAGCMGCCGCGGTAA-3′) and 806R (5′-GGACTACHVGGGTWTCTAAT-3′). All crucial steps during sample processing, DNA isolation and the entire PCR set up were performed in a laminar air flow bench, illuminated with a UV lamp prior to use in order to avoid possible contaminants. The amplicons were normalized, pooled and sequenced on the Illumina MiSeq platform according to the manufacturer’s instructions.

### Accession Codes

The 16S rDNA V4 amplicon sequencing data have been deposited in the NCBI Short Read Archive (SRA) database with accession (the details will be uploaded later).

### Biochemical Data of SFTS Patients

Serum samples were measured for alanine transaminase (ALT), aspartate transaminase (AST) on an Architect C-8000 (Abbott Laboratories, United States) automated chemistry analyzer. All reagents except quality control were provided by Abbott Laboratories. ALT and AST data normal range are 21–72 U/L and 17–59 U/L, respectively. Creatine kinase (CK) and lactate dehydrogenase (LDH) were, respectively, measured with *N*-acetyl-L-cysteine method and the oxidation of lactate on pyruvate method. The normal range was 55–170 U/L and 313–618 U/L, respectively.

### Bioinformatics Analysis and Statistical Analysis

Overlapping paired-end reads form original DNA fragments were been filtered to obtain clean reads by using QIIME (version 1.17) as following (Fadrosh et al., 2014): (1) truncation of sequence reads not having an average quality of 20 over a 30 bp sliding window based on the phred algorithm, and trimmed reads having less than 75% of their original length, as well as its paired read, will be removed; (2) removal of reads contaminated by adapter (default parameter: 15 bases overlapped by reads and adapter with maximal 3 bases mismatch allowed); (3) removal of reads with ambiguous basa (N base), and its paired reads; (4) removal of reads with low complexity (default: reads with 10 consecutive same base). Then, to get tags, clean reads were paired using FLASH (version 1.2.11) as follows (Magoč and Salzberg, 2011): (1) minimal overlapping length: 15 bp; (2) mismatching ratio of overlapped region: ≤0.1; (3) removal of paired end reads without overlaps.

The tags were clustered to Operational Taxonomic Unit (OTUs) by utilizing the software USEARCH (v7.0.1090), detailed as follows: (1) the tags were clustered into OTU with a 97% threshold by using UPARSE (Edgar, 2013); (2) chimeras were filtered out by using UCHIME (v4.2.40) (Edgar et al., 2011). Next, the OTU representative sequences were taxonomically classified using Ribosomal Database Project (RDP) Classifier v.2.2, using 0.8 confidence values as cutoff.

To describe the alpha diversity, Chao 1 index, the Observed species value (the number of OTUs) and Shannon diversity index were calculated. Chao1 index and Observed species which indicated the species richness of community were calculated using R package “fossil,” and Shannon diversity index which indicated the species diversity of the community were calculated using R package “diversity.” Principal component analysis (PCA) was used to display the differences of OTU composition in different samples using R program (v3.1.1).

Continuous variables were reported as means ± standard deviations (SD), and comparisons were made with the Independent *t*-test. For variables that were not normally distributed were reported as Inter-Quartile Range (IQR), comparisons were made with the Mann–Whitney *U* test. In addition, Spearman test was applied to analyze correlation between the clinical variables and gut microbiome. *P*-value of <0.05 was considered statistically significant. All data were analyzed by SPSS (version 20.0; SPSS Inc., Chicago, IL, United States).

## Results

### Cohort Description and Sequencing Data

**FIGURE 1:**
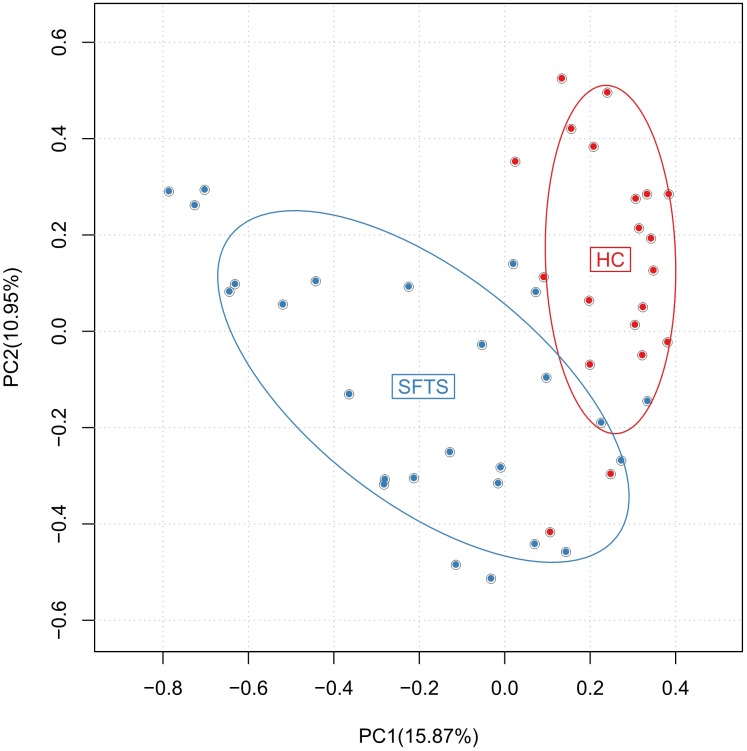
Differences in gut microbiome composition across SFTS and health control groups were assessed by PCA.

Altogether, 46 subjects (SFTS = 26, HC = 20) were enrolled in this study, **Table 1** displays the baseline characteristics of the participants in each cohort. We obtained 3,056,284 overlapping paired-end sequences from the 46 samples, after filtering and removing the chimeric sequences, we obtained 2,894,352 clean reads for further analysis. And then, the clean reads were combined to tags based on overlaps. 1,442,505 tags were obtained in total with a mean of 3,1358 tags per sample, and the mean read length is 252 bp. A total of 648 Operational Taxonomic Units (OTUs) were obtained by clustering the 1,442,505 tags at 97% similarity. The number of OTUs among the SFTS and HC groups were 572 and 472, respectively, and 60.8% of the OTUs (394 OTUs) were overlapped in group SFTS and healthy controls. PCA were used to compare the difference of microbial composition among the SFTS and HC groups, despite the inter-individual variability, **Figure 1** showing the significant separation between the SFTS group and health control groups. The Observed species value, Chao1 index and Shannon index were significantly lower in the SFTS compared to in HC (**Table 2**), indicating that richness and diversity estimators in SFTS are much lower than in HC.

### Variations in Microbial Compositions

**FIGURE 2:**
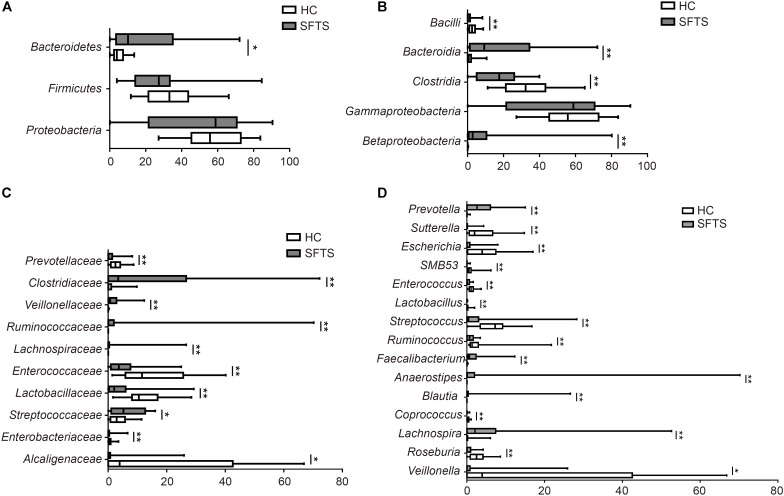
Taxonomic differences of gut microbiota between SFTS, health control groups. Comparison of relative abundance at the bacterial phylum **(A)**, class **(B)**, family **(C)**, genus **(D)** between the two groups. ^∗^*P* < 0.05, ^∗∗^*P* < 0.01.

The gut microbiome from 46 fecal samples belonging to 118 different genera in 14 different phyla. Further analysis of microbiome data showed that SFTS was associated with changes in the fecal microbiota at the phylum, class, family and genus level. At the phylum level, *Bacteroidetes, Firmicutes*, and *Proteobacteria* dominated the fecal microbial communities of both groups (percentage standard deviation of the three major phyla *Bacteroidetes, Firmicutes*, and *Proteobacteria*, were 48.16%, 27.27%, 10.16% for the SFTS, respectively, and 58.57%, 33.20%, 3.95% for health controls). Compared with healthy controls, SFTS patients had fewer *Bacteroidetes* and *Firmicutes*, but higher level of *Proteobacteria* compared with health controls (**Figure 2A**). Additionally, there are more significant difference between SFTS and health controls were discovered at microbial genera level by using univariate analyses.

#### *Bacteroidetes* 

Whatever in the SFTS group or the health controls, the predominant phylum was *Bacteroidetes*, and numerically lower percentage of *Bacteroidetes* were found in SFTS group compared to health controls. And *Bacteroidia* (order *Bacteroidales*) in SFTS group were also numerically lower than in health controls. Further analysis found that although in the same class, different families had different situations. As the dominant family of *Bacteroidetes*, the differences of *Bacteroidaceae* family between two groups were not obvious (**Figure 2C**). However, SFTS patients had much lower level of *Prevotellaceae* family than healthy people, which was attributed to the decrease of *Prevotella* (**Figure 2D**).

#### *Firmicutes* 

Numerically lower percentages of *Firmicutes* were observed in SFTS patients than in healthy controls. At the class level, *Clostridia* (order *Clostridiales*) were significantly reduced in SFTS patients than in health controls, but *Bacilli* (order *Lactobacillales*) were obviously increased in SFTS patients. The predominant bacterial families in the class *Clostridia* were *Lachnospiraceae, Ruminococcaceae, Veillonellaceae*, and *Clostridiaceae*. The *Lachnospiraceae* and *Ruminococcaceae* families which are the representative types of putative butyrate-producing anaerobic bacteria were dramatically decreased in the SFTS group. Compared to health controls, SFTS patients had less *Lachnospiraceae*. And these results were partly due to significant differences in *Lachnospira, Roseburia, Blautia, Coprococcus*, and *Anaerostipes*. *Ruminococcaceae* were also discovered to be significantly underrepresented in SFTS patients compared with healthy controls (**Figure 2D**). Further analysis of this family showed that two genera, *Faecalibacterium* and *Ruminococcus*, led to the differences. On contrast, obviously higher percentages of *Veillonellaceae* family were observed in SFTS patients than in healthy controls (**Figure 2C**), which caused by the increase Veillonella. Although the proportion of *Clostridiaceae* family were both low in the two groups, the SFTS patients had less *Clostridiaceae* than health controls, especially SMB53. In addition, the increase of *Bacilli* class (order *Lactobacillales*) in SFTS patients was attributed to the presence of lactic acid-producing bacteria families such as *Streptococcaneae* family, *Enterococcaceae* and *Lactobacillaceae*, especially *Streptococcus, Lactobacillus*, and *Enterococcus* (**Figure 2D**).

#### *Proteobacteria* 

The abundance of *Proteobacteria* was significantly higher in SFTS patients than in healthy controls. For the SFTS patients, the predominant class of *Proteobacteria* phylum was *Gammaproteobacteria*, whereas the dominant phylum in the health subjects was *Betaproteobacteria*. At the *Betaproteobacteria* class, the *Alcaligenaceae* family was higher in health people than in SFTS patients (**Figure 2B**), which was induced by the increase of *Sutterella*. On the other way, the higher level of *Gammaproteobacteria* in SFTS patients was caused by the increase of *Enterobacteriaceae* family, especially *Escherichia*, which are usually considered as potential opportunistic pathogens (**Figure 2D**).

**Table 2:** The richness and diversity estimators in HC and SFTS groups.

Parameter	SFTS^a^	HC^a^
Observed species value^b∗^	121.19 ± 59.02	167.70 ± 38.11
Chao 1^b∗^	162.40 ± 66.74	205.32 ± 44.02
Shannon index^b∗^	2.43 ± 0.71	2.93 ± 0.57

*^*a*^SFTS, severe fever with thrombocytopenia syndrome; HC, health control. ^*b*^Date are expressed as the mean ± SD. Observed species value (the number of OTUs), the species richness parameter (Chao1) and the species diversity parameter (Shannon index) were calculated using QIIME software. Independent t-test was used to test for significant difference in each variable among two groups. ^∗^Significant difference (p < 0.05) between SFTS and HC groups.*

### Correlation Between Clinical Characteristics and Gut Microbiota

In this study, a total of 26 patients with SFTS were included, 4 (15.38%) patients died. SFTSV-infected patients had acute issue damage as indicated by multiple elevated key serum enzymes, as shown in **Table 1**, ALT, AST, LDH, and CK were all highly elevated in SFTS patients. Our analysis further demonstrated the correlation between gut microbiota and serum enzymes (**Table 3**). Notably, *Lachnospira* showed strong positive association with serum ALT and serum AST, respectively. Interestingly, *Prevotella* and *Lactobacillus* exhibited only moderate positive association with serum ALT. On the contrary, *Ruminococcus* manifested moderate negative association with serum CK. And gut microbiomes showed no relationship between serum LDH.

**Table 3:** Correlation between clinical characteristics and gut microbiota.

Genus^b^	ALT^a^	AST^a^	LDH^a^	CK^a^	Lymphadenopathy	Diarrhea	Vomiting	Infection	Mortality
*Prevotella*	**0.39^∗^**	0.37	-0.04	-0.30	-0.10	0.12	0.28	0.23	0.31
SMB53	0.16	0.34	0.18	-0.06	-0.24	0.05	0.06	0.17	0.18
*Veillonella*	0.19	0.21	0.20	-0.07	0.05	0.24	0.12	**0.46^∗^**	0.21
*Ruminococcus*	-0.09	-0.11	-0.31	-**0.40^∗^**	0.01	0.05	-0.12	0.10	-0.03
*Faecalibacterium*	0.16	0.23	0.20	-0.11	0.31	0.03	0.16	0.32	0.37
*Anaerostipes*	-0.01	0.19	0.17	0.21	0.25	-0.06	-0.09	-0.10	-0.23
*Blautia*	0.13	0.21	0.12	-0.07	0.05	0.11	0.08	0.08	0.04
*Coprococcu*	0.22	0.12	-0.06	-0.12	-0.30	0.03	-0.01	-0.02	0.02
*Lachnospira*	**0.66^∗∗^**	**0.56^∗∗^**	0.16	-0.09	-**0.46^∗^**	0.11	0.05	0.34	0.25
*Roseburia*	0.16	0.25	0.08	-0.07	-0.03	0.15	0.02	0.08	0.07
*Enterococcu*	-0.19	-0.19	-0.17	0.01	0.12	-0.12	-0.14	-0.04	0.10
*Lactobacillus*	**0.42^∗^**	0.35	-0.20	-0.08	-**0.57^∗∗^**	0.23	0.31	0.09	0.20
*Streptococcus*	0.14	0.24	0.12	-0.20	-0.05	0.12	**0.41^∗^**	0.21	**0.48^∗^**
*Escherichia*	0.25	0.30	0.27	0.20	0.02	-0.15	0.08	0.25	0.03
*Sutterella*	0.90	-0.12	-0.13	-0.26	-0.01	0.10	-0.16	0.14	0.07

*^*a*^ALT, alanine transaminase, AST, aspartate transaminase, CK, creatine kinase, LDH, lactate dehydrogenase. ^*b*^The correlation between clinical characteristics and gut microbiota had been analyzed at the bacterial genus level. *^∗∗∗^Correlation between clinical characteristics and gut microbiota at the genus level in SFTS patients, r represents Spearman correlation coefficient, with r value of 0–0.3, 0.3–0.5, and >0.5 indicating weak, moderate and strong correlation between the two variables, respectively. ^∗^ and ^∗∗^ represents P < 0.05 and P < 0.01, respectively**.

For SFTS patients, as its name described, patients suffered high fever and thrombocytopenia. Besides, the clinical characteristics include lymphadenopathy, diarrhea, vomiting, infections, and so on. As shown in **Table 3**, *Lactobacillus* and *Lachnospira* showed strong and moderate negative correlation between lymphadenopathy in SFTS patients, respectively. Since *Streptococcus* are considered as a common opportunistic pathogen, we found that *Streptococcus* exhibited positive association between vomiting and death, respectively. In addition, *Veillonella* showed positive correlation with bacterial infections in SFTS patients. In conclusion, the data suggest that the clinical characteristics were related with changes of gut microbiota in SFTS patients.

## Discussion

Severe fever with thrombocytopenia syndrome is an emerging infectious disease characterized with high fever, thrombocytopenia, leukocytopenia, gastrointestinal symptoms, hemorrhage, and multiple organ failure, with high fatalities ranging from 12 to 30% (Li et al., 2014; Song et al., 2017). In recent years, the number of SFTS cases have increased considerably in China from 511 in 2011 to nearly 1,500 cases in 2012 (Silvas and Aguilar, 2017). SFTS imposes great challenge to the public health because its increased prevalence and limited clinical interventions (Zhang et al., 2012). In this study, the mean age of SFTS patients were 59 years. And a previous research had reported that the majority of SFTS (86%) are detected in subjects 50 years of age or older (Liu et al., 2015), which is same with our finding. Previous studies had pay attention to the ecology and transmission cycle (Wang et al., 2015; Li et al., 2016), pathogenesis (Wu et al., 2014; Ning et al., 2015; Silvas et al., 2015) and treatment (Kim et al., 2016; Yoo et al., 2017) of SFTS, but no one has pointed to the alteration of gut microbiota for SFTS patients, this study aim to find some differences of gut microbiome between SFTS patients and health people.

Severe fever with thrombocytopenia syndrome appears to decrease intestinal microbiome richness as those in SFTS patients had less number of OTUs and lower Chao 1 index compared to those in the health controls. And the Shannon index for SFTS patients was lower than it for health controls indicated that SFTS appears to decrease the diversity of the gut microbiota. The results were similar at both the phylum and genus level. Cotillard et al. (2013) had illustrated that obesity not only influenced the by the numbers and richness of gut microbiota and the diversity of gut microbiome. According to this, we speculate that the richness and diversity of gut microbiome both have some influence on pathogenesis of SFTS, and the specific mechanism needs further study.

In the human intestinal microbiome, *Bacteroidetes* and *Firmicutes* are the dominant bacteria, which accounting for approximately 99% of the whole bacteria (Li et al., 2008). However, in our study, at the phylum level, the relative abundance of *Bacteroidetes* and *Firmicutes* were numerically lower in SFTS patients, but the proportion of *Proteobacteria* was significantly higher in SFTS than healthy people. Different from previous studies, which had found that fat people had lower *Firmicutes* and higher *Bacteroidetes* (Zhu et al., 2013) and liver cirrhosis patients had less *Bacteroidetes* and more *Proteobacteria* compared with health people (Qin N. et al., 2014). The novelty of our study is that we provide the first report of a significant alteration of intestinal microbiota of SFTS patients compared with healthy subjects. In our research, the higher *Proteobacteria* and lower *Bacteroidetes* and *Firmicutes* showing a severe ratio of “bad vs. good” taxa abundance associated with SFTS. Previous study had suggested that *Bacteroidetes* and *Firmicutes* were increased after successful fecal microbial transplantation (FMT), and *Proteobacteria* were less abundant than before (Hamilton et al., 2013).

At the family level, the decrease of the phylum *Firmicutess* in SFTS patients was mostly associated with the families *Lachnospiraceae* and *Ruminococcaceae*, which were the predominant members of *Clostridia* class, usually considered as ‘healthy’ gene-rich microbiota as it has anti-inflammatory properties. These bacteria, as typical representatives of short-chain fatty acids (SCFAs)-producing bacteria, play a key role in producing nutrients for the host and supplying energy for the colonic epithelium, regulating host gene expression, inflammation and differentiation (Canani et al., 2011). A decrease of SCFAs would result in deteriorated intestinal integrity and increased intestinal permeability, which may cause worse gastrointestinal symptoms in SFTS. The SMB53 which belong to *Clostridiaceae* family in *Clostridia* class were also reduced in SFTS patients. Besides the reduction of *Lachnospiraceae* and *Ruminococcaceae*, there were also some common opportunistic pathogens significantly increased in SFTS patients, including *Enterococcaceae* and *Streptococcaceae*, especially *Enterococcus* and *Streptococcus*. However, the increase of *Enterococcaceae* and *Streptococcaceae* could be a result, or a cause of progression in SFTS, and further studies are necessary to assess the causal relationship. Meanwhile, *Lactobacillaceae* family and its most abundant genus *Lactobacillus* were clearly increased in SFTS patients compared with health people. Antharam et al. (2013) had also revealed an increase of *Lactobacillus* in antibiotic-associated diarrhea compared with healthy people. Actually, *Lactobacillaceae* family, and the bacteria family *Enterococcaceae* and *Streptococcaceae*, belong to the same order *Bacilli*, and all of these are good at producing lactic acid. We speculate that besides the increasing of *Enterococcaceae* and *Streptococcaceae* may lead more risk of opportunistic infections of SFTS patients, the function of producing lactic acid by these bacteria especially *Lactobacillus* may also paly indeed role in the progression of SFTS. It is worth noting that a recent study had reported that the reduction of *Lactobacillus* spp. caused by high salt diet could contribute to hypertension, which by increasing T helper 17 cells (Wilck et al., 2017). Interestingly, Ritze et al. (2014) demonstrated that *Lactobacillus* could attenuate liver pathologies through anti-inflammatory actions and stabilization of the intestinal barrier. The inevitable discrepancies among different studies may due to the different diseases and host factors influencing the intestinal microbiome. In addition, we firstly found that *Veillonellaceae* were much higher in SFTS patients compared with healthy controls. The finding was in accordance with a previous study which reported that liver cirrhosis had more *Veillonellaceae* which came from oral (Qin N. et al., 2014). We suspect the origin of *Veillonellaceae* in the gut microbiota of SFTS patients, but it needs further validation.

Our present study found that the higher proportion of *Proteobacteria* was contributed by the increase of lipopolysaccharide-producing G-bacteria, such as *Enterobacteriaceae* family, especially *Escherichia*. The higher abundance of these opportunistic pathogens would may result in endotoxemia in SFST patients. Differ from the increase of *Enterobacteriaceae*, we found that the *Sutterella* in *Alcaligenaceae* family was reduced in SFTS patients. Morgan et al. (2015) had reported that host inflammation processes were inversely correlated with *Sutterella* and positively correlated with *Escherichia* in inflammatory bowel disease. We guess that these bacteria may have same influence on SFST which need to confirm. A previous study had found that high level of *Prevotella* was associated with the development of hypertension and hyperlipidemia (Liu F. et al., 2017), which contrary in our study, the *Prevotella* was reduced in the SFTS patients. Furthermore, a former research had revealed that the content of *Prevotella* was same in patients with non-alcoholic fatty liver disease compared with healthy people (Wang et al., 2016). It demonstrated that the alteration of gut microbiota in multiple diseases have its own characteristics.

There have been a limited number of studies in detail on the correlation between gut microbiota and clinical characteristics after SFTSV infection. In accordance with our study *Streptococcaceae* found to be significantly associated with vomiting and mortality of SFTS patients. The higher abundance of *Streptococcaceae* which are opportunistic pathogens, may be a consequence of imbalance in microbiota resulting in the fetal outcome of SFTS patients. In fact, in SFTS patients, *Veillonellaceae* were related with bacterial infections. Indeed, previous study had reported that SFTS patients had a higher risk of secondary bacterial infection, which resulted in an increased morality (Liu et al., 2014). The link between *Veillonellaceae* and bacterial infections indicate that the *Veillonellaceae* may contribute to the aggravation of SFTS, but it needs further validation. In this study, *Lactobacillus* had showed strong negative correlation between lymphadenopathy and moderate positive association with serum ALT. As we all know that the function of *Lactobacillus* was producing lactic acid, but the role of *Lactobacillus* in the pathogenesis of SFTS needs further analysis. *Prevotella* manifested positive association with serum ALT, and the specific mechanism needs further study. Meanwhile, *Lachnospiraceae* and *Ruminococcaceae*, which were the predominant members of SCFAs-producing bacteria, exhibited different relationship with clinical characteristics in SFTS patients. For *Ruminococcaceae*, it showed significant negative correlation with serum CK. One of the characteristic laboratory findings in the severity SFTS was higher serum CK (Liu J. et al., 2017). We think that decreased *Ruminococcaceae* may play an important role in the pathogenesis of SFTS, and increased the level of *Ruminococcaceae* may be a new idea for the therapy for SFTS patients. On the contrary, we observed the phenomenon that *Lachnospiraceae* were strong positive related with ALT and AST and negative associated with lymphadenopathy. We feel confused about the causes of this phenomenon and will continue to explore it in subsequent research.

## Conclusion

Our present study firstly demonstrated that the gut microbiota of SFTS patients were significant different with health people. Not only the richness and diversity of intestinal microbiome were reduced in SFTS, but also the composition of it had significant differences. The good bacteria such as *Lachnospiraceae* and *Ruminococcaceae* which could produce short-chain fatty acids were clearly dropped in SFTS. Meanwhile, *Sutterella* which have anti-inflammation properties were reduced too. On the contrary, some common opportunistic pathogens like *Enterococcus* and *Streptococcus* which could rise the risk of infections were increased in SFTS patients than healthy people, and high level of lactate-producing bacteria *Lactobacillaceae* could take a heavy on the development of SFTS. Moreover, the increase of endotoxin-producing bacteria *Escherichia* also adversely affected the SFTS patients. Besides, the analysis of the correlations between clinical index and gut microbiota indicated that gut microbiota was strong associated with key serum enzymes level, lymphadenopathy, vomiting, infection and mortality in SFTS. So far, the evidence supporting the role of intestinal dysbiosis in SFTS is limited, and the impact of some changes of bacteria in SFTS are still confused, further studies underlying the regulatory mechanisms and functions of gut microbiome are essential.

## Author Contributions

HX and YW performed the laboratory work. YW performed the data analysis. HX wrote the manuscript. JL and LH provided ideas and checked the manuscript. HM, YL, and YZ provided suggestions.

## Conflict of Interest Statement

The authors declare that the research was conducted in the absence of any commercial or financial relationships that could be construed as a potential conflict of interest.
