# Corrigendum: Alterations of Gut Microbiome in the Patients With Severe Fever With Thrombocytopenia Syndrome

**DOI:** 10.3389/fmicb.2019.00251

**Published:** 2019-02-19

**Authors:** Honghai Xu, Yuanyuan Wei, Hongqiu Ma, Yanyan Liu, Yalong Zhang, Lifen Hu, Jiabin Li

**Affiliations:** ^1^Department of Infectious Diseases, The First Affiliated Hospital of Anhui Medical University, Hefei, China; ^2^Department of Pathology, The First Affiliated Hospital of Anhui Medical University, Hefei, China; ^3^Anhui Center for Surveillance of Bacterial Resistance, Hefei, China; ^4^Department of Hospital Infection Control, The First Affiliated Hospital of Anhui Medical University, Hefei, China

**Keywords:** sever fever with thrombocytopenia syndrome (SFTS), gut microbiome, 16S rDNA sequencing, *Bacteroidetes*, *Firmicutes*, *Proteobacteria*, clinical symptoms, key serum enzymes

In the original article, there was a mistake in Figure 2 as published. In this article, Figure 2 is used to introduce the taxonomic differences of gut microbiota between SFTS and healthy control groups. According to the reviewers' helpful comments, we tried to integrate one chart and three tables from the original manuscript into one new figure. However, there are some misalignments in the label of the Y-axis in Figure 2. When integrating these four images (A, B, C, and D) into Figure 2, the order of the Y-axis annotations of each graph were reversed. The corrected Figure 2 appears below.

**Figure 2:**
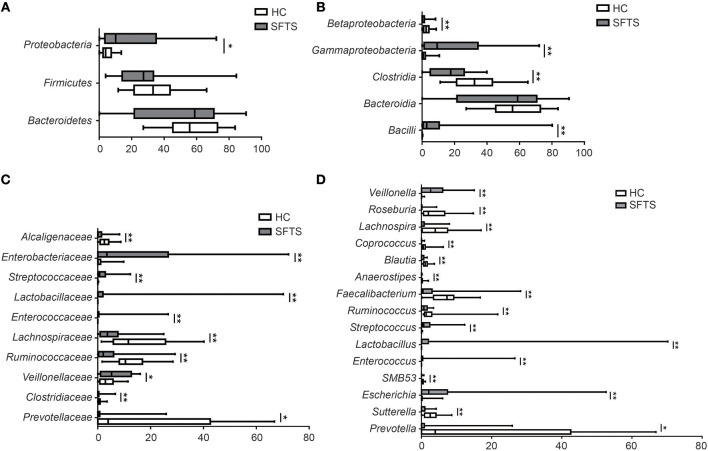
Taxonomic differences of gut microbiota between SFTS, health control groups. Comparison of relative abundance at the bacterial phylum **(A)**, class **(B)**, family **(C)**, genus **(D)** between the two groups. ^*^P < 0.05, ^**^P < 0.01.

The authors apologize for this error and state that this does not change the scientific conclusions of the article in any way.

## Conflict of Interest Statement

The authors declare that the research was conducted in the absence of any commercial or financial relationships that could be construed as a potential conflict of interest.

