# Supplementary material for: Alterations of Gut Microbiome in the Patients With Severe Fever With Thrombocytopenia Syndrome
Source: Front Microbiol. 2018 Oct 1;9:2315. doi: 10.3389/fmicb.2018.02315 (PMC6174290; doi:10.3389/fmicb.2018.02315)
Supplement: Supplementary file 1 [file Table_1.DOCX]

Supplementary Table 1 Baseline characteristics of subjects

| Numbers | Gender^c^ | Ages | Region^d^ | ALT^e^  U/L | AST^f^  U/L | LDH^g^  U/L | CK^h^  U/L | lymphadenopathy^i^ | diarrhea | vomiting | infection | mortality |
| --- | --- | --- | --- | --- | --- | --- | --- | --- | --- | --- | --- | --- |
| HC^a^-1 | M | 39 | Lu’an | 25 | 22 | 203 | 121 | 0 | 0 | 0 | 0 | 0 |
| HC-2 | M | 42 | Anqing | 38 | 29 | 218 | 140 | 0 | 0 | 0 | 0 | 0 |
| HC-3 | M | 51 | Anqing | 10 | 23 | 102 | 116 | 0 | 0 | 0 | 0 | 0 |
| HC-4 | M | 52 | Lu’an | 25 | 21 | 187 | 111 | 0 | 0 | 0 | 0 | 0 |
| HC-5 | M | 55 | Lu’an | 25 | 22 | 149 | 73 | 0 | 0 | 0 | 0 | 0 |
| HC-6 | M | 58 | Lu’an | 10 | 23 | 185 | 71 | 0 | 0 | 0 | 0 | 0 |
| HC-7 | M | 65 | Anqing | 23 | 21 | 144 | 89 | 0 | 0 | 0 | 0 | 0 |
| HC-8 | M | 68 | Lu’an | 24 | 23 | 207 | 98 | 0 | 0 | 0 | 0 | 0 |
| HC-9 | M | 70 | Lu’an | 12 | 18 | 156 | 69 | 0 | 0 | 0 | 0 | 0 |
| HC-10 | F | 43 | Anqing | 10 | 21 | 179 | 60 | 0 | 0 | 0 | 0 | 0 |
| HC-11 | F | 43 | Lu’an | 11 | 20 | 205 | 81 | 0 | 0 | 0 | 0 | 0 |
| HC-12 | F | 44 | Anqing | 20 | 25 | 187 | 46 | 0 | 0 | 0 | 0 | 0 |
| HC-13 | F | 47 | Anqing | 30 | 24 | 190 | 153 | 0 | 0 | 0 | 0 | 0 |
| HC-14 | F | 52 | Lu’an | 26 | 24 | 195 | 91 | 0 | 0 | 0 | 0 | 0 |
| HC-15 | F | 55 | Lu’an | 28 | 28 | 263 | 79 | 0 | 0 | 0 | 0 | 0 |
| HC-16 | F | 59 | Anqing | 23 | 38 | 266 | 80 | 0 | 0 | 0 | 0 | 0 |
| HC-17 | F | 61 | Lu’an | 7 | 25 | 217 | 175 | 0 | 0 | 0 | 0 | 0 |
| HC-18 | F | 63 | Lu’an | 26 | 33 | 234 | 76 | 0 | 0 | 0 | 0 | 0 |
| HC-19 | F | 67 | Lu’an | 9 | 20 | 224 | 52 | 0 | 0 | 0 | 0 | 0 |
| HC-20 | F | 73 | Anqing | 45 | 56 | 254 | 89 | 0 | 0 | 0 | 0 | 0 |
| SFTS^b^-1 | M | 49 | Lu’an | 319 | 297 | 2150 | 260 | 0 | 0 | 1 | 1 | 1 |
| SFTS-2 | M | 49 | Anqing | 63 | 110 | 1102 | 731 | 1 | 1 | 0 | 1 | 0 |
| SFTS-3 | M | 51 | Lu’an | 109 | 81 | 1216 | 302 | 0 | 0 | 0 | 0 | 0 |
| SFTS-4 | M | 53 | Lu’an | 251 | 746 | 5673 | 9440 | 1 | 0 | 0 | 0 | 0 |
| SFTS-5 | M | 58 | Anqing | 28 | 49 | 679 | 136 | 1 | 1 | 0 | 0 | 0 |
| SFTS-6 | M | 59 | Anqing | 513 | 750 | 3606 | 1600 | 0 | 0 | 1 | 1 | 1 |
| SFTS-7 | M | 61 | Lu’an | 73 | 220 | 1851 | 6003 | 1 | 0 | 0 | 0 | 0 |
| SFTS-8 | M | 64 | Lu’an | 39 | 136 | 1435 | 511 | 0 | 0 | 0 | 0 | 0 |
| SFTS-9 | M | 66 | Lu’an | 155 | 392 | 2150 | 1580 | 1 | 0 | 0 | 1 | 0 |
| SFTS-10 | M | 67 | Lu’an | 128 | 91 | 362 | 504 | 0 | 0 | 0 | 0 | 0 |
| SFTS-11 | M | 67 | Lu’an | 239 | 368 | 940 | 661 | 1 | 1 | 0 | 0 | 0 |
| SFTS-12 | M | 73 | Anqing | 80 | 179 | 908 | 112 | 0 | 1 | 0 | 0 | 0 |
| SFTS-13 | M | 73 | Anqing | 36 | 64 | 857 | 89 | 1 | 1 | 1 | 1 | 1 |
| SFTS-14 | F | 41 | Anqing | 71 | 253 | 890 | 109 | 0 | 1 | 0 | 0 | 0 |
| SFTS-15 | F | 47 | Anqing | 376 | 360 | 1012 | 160 | 0 | 0 | 0 | 1 | 0 |
| SFTS-16 | F | 49 | Lu’an | 40 | 47 | 593 | 75 | 1 | 0 | 0 | 0 | 0 |
| SFTS-17 | F | 53 | Lu’an | 53 | 51 | 1793 | 163 | 1 | 0 | 0 | 1 | 0 |
| SFTS-18 | F | 54 | Lu’an | 406 | 641 | 2150 | 129 | 1 | 1 | 1 | 0 | 0 |
| SFTS-19 | F | 54 | Anqing | 79 | 105 | 796 | 93 | 1 | 1 | 1 | 0 | 0 |
| SFTS-20 | F | 60 | Lu’an | 45 | 89 | 1593 | 206 | 1 | 1 | 1 | 1 | 0 |
| SFTS-21 | F | 62 | Lu’an | 95 | 329 | 1498 | 1160 | 1 | 0 | 0 | 0 | 0 |
| SFTS-22 | F | 65 | Lu’an | 83 | 87 | 381 | 159 | 1 | 0 | 0 | 0 | 0 |
| SFTS-23 | F | 67 | Anqing | 670 | 568 | 4489 | 208 | 0 | 0 | 0 | 0 | 0 |
| SFTS-24 | F | 69 | Anqing | 37 | 51 | 918 | 399 | 0 | 1 | 1 | 1 | 1 |
| SFTS-25 | F | 71 | Anqing | 772 | 145 | 401 | 84 | 0 | 0 | 0 | 0 | 0 |
| SFTS-26 | F | 71 | Xinyang | 80 | 219 | 2150 | 1169 | 1 | 0 | 0 | 0 | 0 |

^a^ HC, health control.

^b^ SFTS, severe fever with thrombocytopenia syndrome.

^c^ M, Male; F, Female.

^d^ regions of SFTS patients and health control group, which all belong to Ta-pieh Mountains.

^e^ ALT, alanine transaminase, the normal range is 21-72 U/L.

^f^ AST, aspartate transaminase, the normal range is 17-59 U/L.

^g^ LDH, lactate dehydrogenase, the normal range is 313-618 U/L.

^h^ CK, creatine kinase, the normal range is 55-170 U/L.

^i^ 0, no corresponding clinical sign; 1, a corresponding clinical sign.
